# Proteomics in Schizophrenia: A Gateway to Discover Potential Biomarkers of Psychoneuroimmune Pathways

**DOI:** 10.3389/fpsyt.2019.00885

**Published:** 2019-11-29

**Authors:** Daniela Rodrigues-Amorim, Tania Rivera-Baltanás, María del Carmen Vallejo-Curto, Cynthia Rodriguez-Jamardo, Elena de las Heras, Carolina Barreiro-Villar, María Blanco-Formoso, Patricia Fernández-Palleiro, María Álvarez-Ariza, Marta López, Alejandro García-Caballero, José Manuel Olivares, Carlos Spuch

**Affiliations:** ^1^Translational Neuroscience Research Group, Galicia Sur Health Research Institute, University of Vigo, CIBERSAM, Vigo, Spain; ^2^Department of Psychiatry, University of Santiago de Compostela, Santiago de Compostela, Spain

**Keywords:** schizophrenia, proteomics, liquid chromatography–tandem mass spectrometry, Rab3 GTPase-activating protein catalytic subunit, glia maturation factor beta, brain-derived neurotrophic factor

## Abstract

Schizophrenia is a severe and disabling psychiatric disorder with a complex and multifactorial etiology. The lack of consensus regarding the multifaceted dysfunction of this ailment has increased the need to explore new research lines. This research makes use of proteomics data to discover possible analytes associated with psychoneuroimmune signaling pathways in schizophrenia. Thus, we analyze plasma of 45 patients [10 patients with first-episode schizophrenia (FES) and 35 patients with chronic schizophrenia] and 43 healthy subjects by label-free liquid chromatography–tandem mass spectrometry. The analysis revealed a significant reduction in the levels of glia maturation factor beta (GMF-β), the brain-derived neurotrophic factor (BDNF), and the 115-kDa isoform of the Rab3 GTPase-activating protein catalytic subunit (RAB3GAP1) in patients with schizophrenia as compared to healthy volunteers. In conclusion, GMF-β, BDNF, and 115-kDa isoform of RAB3GAP1 showed significantly reduced levels in plasma of patients with schizophrenia, thus making them potential biomarkers in schizophrenia.

## Introduction

As a major psychiatric disorder, schizophrenia is characterized by a particular symptomatology that includes both positive and negative symptoms, as well as cognitive impairment (1). Schizophrenia affects approximately 1% of the worldwide population and is a devastating disease that requires a large amount of medical resources owing to its early onset and chronicity (2). Therefore, its timely recognition and intervention enables the prevention of psychosis onset, as well as a reduction in the severity of the disease (3, 4). Schizophrenia presents with dissimilar clinical phenotypes whose heterogeneity hampers the consolidation of its pathophysiology (5, 6). The high complexity that characterizes schizophrenia may be explained by a combination of genetic and environmental factors (7, 8).

New research lines and approaches using proteomic techniques have recently arisen in search of a new explanation for the pathogenesis of schizophrenia (9, 10). Proteomics provides a molecular vision of the ailment that supports the acquisition of new knowledge about its biochemical pathways and the identification of potential biomarkers. This "omics" science recognizes a functional profile of proteins expressed in the proteome of individuals by means of high-throughput methods such as liquid chromatography–tandem mass spectrometry (LC-MS/MS) (8, 11). As a multifaceted disease, schizophrenia involves changes in protein expression, thus also affecting protein networks and signal pathways (9). These alterations can be detected by proteomic analyses performed on peripheral samples such as plasma. Of particular interest is the discovery of new plasma biomarkers associated with neuronal alterations (12, 13). One of the handicaps of the discovery of new analytes related to mental disorders, such as schizophrenia, is the need to work with peripheral samples to detect potential brain damages. To increase the likelihood of detecting new proteins, we analyzed plasma levels using shotgun proteomic techniques (LC-MS/MS) (14).

In this study, the proteome analysis allowed the discovery of potential new analytes whose specificity was then evaluated with immunoassays. For our research, we carried out a proteomic study on plasma samples of patients with schizophrenia in comparison with that of healthy subjects, obtaining promising findings in the identification of proteins as potential biomarkers of schizophrenia.

## Material and Methods

### Human Samples

Venous blood samples from 88 participants, 45 patients diagnosed with schizophrenia [10 patients with first-episode schizophrenia (FES) and 35 patients with chronic schizophrenia] and 43 health controls were collected in vacuum tubes containing dipotassium ethylenediaminetetraacetic acid (K_2_EDTA) between 7:00 and 9:00 hours. To check the presence of these analytes in the cerebrospinal fluid (CSF), we also obtained six CSF human samples from healthy controls by lumbar puncture. The recruitment period lasted 8 months, and samples of these patients and control subjects were obtained from the Álvaro Cunqueiro Hospital in Vigo, Spain. The diagnosis of schizophrenia was reached by psychiatrists based on the fifth edition of the *Diagnostic and Statistical Manual of Mental Disorders* (*DSM-5*) and on the most common terminology used in schizophrenia studies and guidelines (15). The study’s inclusion criteria were patients with schizophrenia aged 18 years or older who provided a signed informed consent compliant with the guidelines of the Helsinki Declaration and approved by the ethics committee (Galician Network of Research Ethics Committees). Subjects with other psychiatric or neurological disorders, a traumatic brain injury, or a history of substance abuse were excluded from the study. Pregnant or breastfeeding women were also excluded. The control group was selected based on the frequency matching method (cases and controls have similar distributions of matching variables). Patient cohorts were matched by age and sex with control group, which was composed of healthy volunteers 18 years of age or older with no psychiatric or neurological illnesses. Moreover, the control group was also requested to provide a signed informed consent.

### Clinical and Demographic Assessment

A demographic questionnaire was administered to all patients and healthy volunteers upon their admission to the Psychiatry Unit of the Álvaro Cunqueiro Hospital. The Positive and Negative Syndrome Scale (PANSS) was also used to evaluate the intensity of their positive and negative symptoms, as well as the general psychopathology of the patients with schizophrenia. Both instruments were performed and assessed by a trained psychologist independent from the study. The clinical and demographic characteristics of the study participants are shown in **Table 2**.

### Blood Sample Preparation

Venous blood collected in two vacuum tubes containing K_2_EDTA (BD vacutainer; Becton, Dickinson & Co., USA) was centrifuged at 1,000 × *g* for 10 min to separate the blood plasma, which was subsequently stored in aliquots at −80 °C. An aliquot of 450 µl of plasma was then centrifuged at 16,000 × *g* for 15 min at 4 °C, and the resulting supernatant was collected and stored at −80 °C. Proteins were measured by means of a bicinchoninic acid (BCA) assay (Pierce Chemicals, Rockford, IL). In addition, a protein enrichment commercial kit (ProteoMiner^™^, Bio-Rad, Hercules, California, USA) was used according to the manufacturer’s instructions to equalize proteins in a sample with a total protein content of 10 mg. Ten microliters of eluted fractions were then loaded onto a standard Laemmli buffer 2x polyacrylamide gel, allowing them to stack and enter the resolving gel, but not to separate. The gel was subsequently cut by hand with a sterile scalpel under a flow chamber, and the excised pieces of gel were subjected to in-gel digestion consisting in washing the gel pieces sequentially with ammonium bicarbonate 25 mM and 50% acetonitrile (ACN)/ammonium bicarbonate 25 mM in an ultrasonic bath. The proteins were later reduced with dithiothreitol (DTT) 10 mM for 1 h and alkylated with indoleacetic acid (IAA) 55 mM for 30 min. Finally, proteins were digested overnight with 40 ng of trypsin at 37 °C, and tryptic peptides were extracted from the gel matrix in two steps with 0.5% trifluoroacetic acid (TFA) and 100% ACN.

### Liquid Chromatography–Tandem Mass Spectrometry

The tryptic peptides were then dried in a speed-vacuum concentrator at 45 °C (Concentrator plus, Eppendorf, Hamburg, Germany), reconstituted in LC/MC-grade water containing 0.1% (v/v) formic acid, and analyzed by electrospray ionization–tandem mass spectrometry in a hybrid high-resolution LTQ-Orbitrap ELITE (Thermo Fisher Scientific, Waltham, MA, USA) coupled to an Easy-nLC 1000 liquid chromatography system (Thermo Fisher Scientific, Waltham, MA, USA). They were subsequently transferred to a reverse phase column (PepMap^®^ RSLC C18, 2 µm, 100 Å, 75 µm x 500 mm, Thermo Fisher Scientific, Waltham, MA, USA) and eluted with an ACN gradient of 5–30% containing 0.1% formic acid, for 240 min and at a flow rate of 5 µl/min. The resulting elutes were transferred directly to the mass spectrometer, which was set to a positive ion setting in a data-dependent mode. A full mass spectrometry scan was performed with a mass-to-charge ratio range of 350–1,600 m/z and a resolution of 120,000. A tandem mass spectrometry scan was then performed with the top 15 at 18% of the normalized collision energy (NCE), with a dynamic exclusion time of 30 s, a minimum signal threshold of 1,000, a resolution of 30,000, and an isolation width of 1.50 Da.

### Search and Analysis of Protein Databases

A bioinformatic analysis of MS/MS raw data was performed using the MaxQuant 1.6.1.0 software (http://www.coxdocs.org/doku.php?id=maxquant:start). Protein identification was carried out using the UniProtKB/Swiss-Prot human proteome (release 2018_04). For the quantitative analysis, we considered an false discovery rate (FDR) <0.01 for proteins with at least two matching unique peptides (17). Searches were performed by applying the following group-specific parameters: trypsin/P was set as the digestion enzyme with a maximum of two missing cleavages, and up to five modifications per peptide were permitted; the methionine (M) oxidation and protein N-terminal acetylation were set as variable modifications; minimal peptide length was set to seven amino acids; and the label-free quantification (LFQ) ratio count was set to two with normalization. Following this workflow, the MaxQuant package was used to create tables in.txt format, which were then analyzed with the Perseus 1.6.1.1 software (http://www.coxdocs.org/doku.php?id=perseus:start) (18). Given that our research hypothesis was based on the specific functions carried out by proteins at the level of the central nervous system (CNS), such as neuronal, synaptic, and neurotransmission processes, the table (proteinGroups.txt) generated by MaxQuant was uploaded in Perseus, and the data were filtered based on categorical columns to remove potential contaminants and reverse hits, and to exclude proteins exclusively identified by site. The LFQ intensities were subsequently transformed into logarithms, and missing data are replaced by imputation of missing values according to a normal distribution (width: 0.3; downshift: 1.8). The Perseus table was finally exported to Excel, where functional proteins were characterized based on their activity, molecular function, biological processes, and location. The UniProt (https://www.uniprot.org/) and Ensemble (https://www.ensembl.org/) databases were also consulted to obtain specific information on the proteins, and the STRING 10.5 online platform was used to identify protein interaction networks.

### Western Blot Analysis

Aliquots of plasma samples of both the patients with schizophrenia and the healthy controls, and aliquots of CSF of six controls were mixed with 2X volume of Laemmli buffer 2X (Bio-Rad, California, USA) (950 µl of Laemmli buffer and 50 µl of β-mercaptoethanol) and boiled at 95 °C for 5 min. Previous measurement of the plasma and CSF proteins (total proteins) allowed for determining the equivalent amount of proteins required for each sample (10 µg of proteins). Fraction samples were loaded in 6–14% Bis-Tris polyacrylamide gels, and an electrophoresis was performed in a PowerPac^™^ universal power supply (Bio-Rad, California, USA) at 60 V for 30 min, and at 100 V for another 90 min. The proteins were immediately transferred to polyvinylidene difluoride membranes (Immun-Blot^®^ polyvinylidene fluoride (PVDF) membrane, Bio-Rad, California, USA) contained in a PowerPac^™^ universal power supply (Bio-Rad, California, USA) set at 0.25 A for 60 min (for two gels). The membranes were blocked with 5% milk in a tris-buffered saline solution with Tween (TBST) for 20 min and washed three times with the same TBST solution. The membranes were then incubated overnight at 4 °C over stirrers with a primary antibody: anti-drebrin (C-8) mouse monoclonal antibody 1:1,000 (sc-374269, Santa Cruz Biotechnology, Inc., OR, USA); anti–glia maturation factor beta (GMF-β) rabbit polyclonal antibody 1:1,000 (PA5-70673, Thermo Fisher Scientific, Rockford, USA); anti–Rab3 GTPase-activating protein catalytic subunit (RAB3GAP1) rabbit polyclonal antibody 1:1,000 (PA5-37026, Thermo Fisher Scientific, Rockford, USA); anti-attractin (9H8) mouse monoclonal antibody 1:1,000 (LF-MA0146, Thermo Fisher Scientific, Rockford, USA); or anti–brain-derived neurotrophic factor (BDNF) rabbit polyclonal antibody 1:2,000 (SAB2108004, Sigma-Aldrich, St. Louis, USA). After washing them three times with TBST, the membranes were incubated with the appropriate secondary antibody 1:10,000 (GE Healthcare Life Sciences, UK) for 60 min over a stirrer. The membranes were then washed again twice with TBST and once with TBS. The ChemiDoc XRS+ system (Bio-Rad, California, USA) was then used to analyze the chemiluminescence of the membranes with the ECL^™^ Prime Western Blotting System (Sigma-Aldrich, St. Louis, USA). Image Lab 6.0 software (Bio-Rad, California, USA) was used to analyze the blot images acquired, and a densitometric band quantification was performed by means of the ImageJ 1.5k software (National Institutes of Health, USA).

### Statistical Analysis

The GraphPad Prism 7 software (GraphPad Software Inc., San Diego, CA, EUA) was used to manage the resulting data and to perform the statistical analysis. The LFQ proteomics analysis was carried out in the Perseus 1.6.1.1 software. A two-sample Student’s *t*-test was used to analyze differences in LFQ protein intensities between the patients with schizophrenia and the controls, and the FDR *Q* values were corrected using the Benjamini–Hochberg method (*Q*-value threshold ≤0.05). The mean age of both cohorts was compared with the Mann–Whitney U test, and the differences between sex ratios were analyzed with Fisher’s exact test. To determine whether there are significant differences in means between schizophrenia subgroups (FES and chronic schizophrenia) and control group it was applied one-way ANOVA followed by Bonferroni post-hoc test. Effect size estimates (Hedges’ g) were calculated using means, standard deviations, and sample size, and were interpreted according to Cohen guidelines, with an effect size of 0.2, 0.5, and 0.8 (small, medium, and large, respectively). In addition, the correlation between the PANSS scores, duration of illness, and age of illness onset, and the relative protein units obtained in both cohorts (schizophrenia and controls) was analyzed by means of a Pearson correlation analysis. Statistically significant results are assumed considering a *P* value ≤ 0.05.

## Results

We performed a case–control study comparing a cohort of patients with schizophrenia with a cohort of health subjects whose parameters closely matched those of the schizophrenia group. Our research hypothesis focused on identifying proteins linked to specific functions carried out at the level of the CNS, such as neuronal (neuroinflammation, neurodegeneration), neurotransmission (exocytosis of neurotransmitters), and synaptic activity (synaptic plasticity). Thus, the design of our proteomics experiment considered both statistical and theoretical principles (19). Plasma proteomics enabled the identification of a large number of proteins as we performed a proteomic analysis with an appropriate workflow including processes such as sample preparation, data analysis, and the assessment of cohesive results with a view to export this new knowledge to the clinical practice. Our comparison of the demographic and clinical data of 45 patients with schizophrenia and 43 healthy controls revealed no significant differences between both cohorts in terms of age (*P* = 0.4593 Mann–Whitney U test) and sex (*P* = 1.0000 Fisher’s exact test). The raw data obtained with the Thermo Orbitrap MS were analyzed in the MaxQuant software, with a total of 1,302 proteins being screened. An essential criterion established for our analysis was that the proteins had to be directly correlated with specific functions in the CNS, in particular relating to psychoneuroimmune signaling pathways. The MaxQuant database (proteinGroups.txt) was converted into an Excel file (XLS) on the basis of this criterion, and a careful analysis of each protein was carried out according to its name, encoding gene, molecular, and biological functions, and subcellular location, using the UniProt database as a reference. Following this analysis, 34 proteins that met our criteria were screened. A two-sample test (Student’s *t*-test, minimum fold-change [S0] = 2; FDR = 0.01) was performed in the Perseus software. The FDR *Q* values were corrected with the Benjamini–Hochberg method (**Supplementary Table 1**) and represented logarithmically (Log10 [FDR *q* value]) with a *Q*-value threshold ≤ 0.05 (**Supplementary Figure 1**).

### Alterations of Psychoneuroimmune Pathways in Patients With Schizophrenia

**Figure 1:**
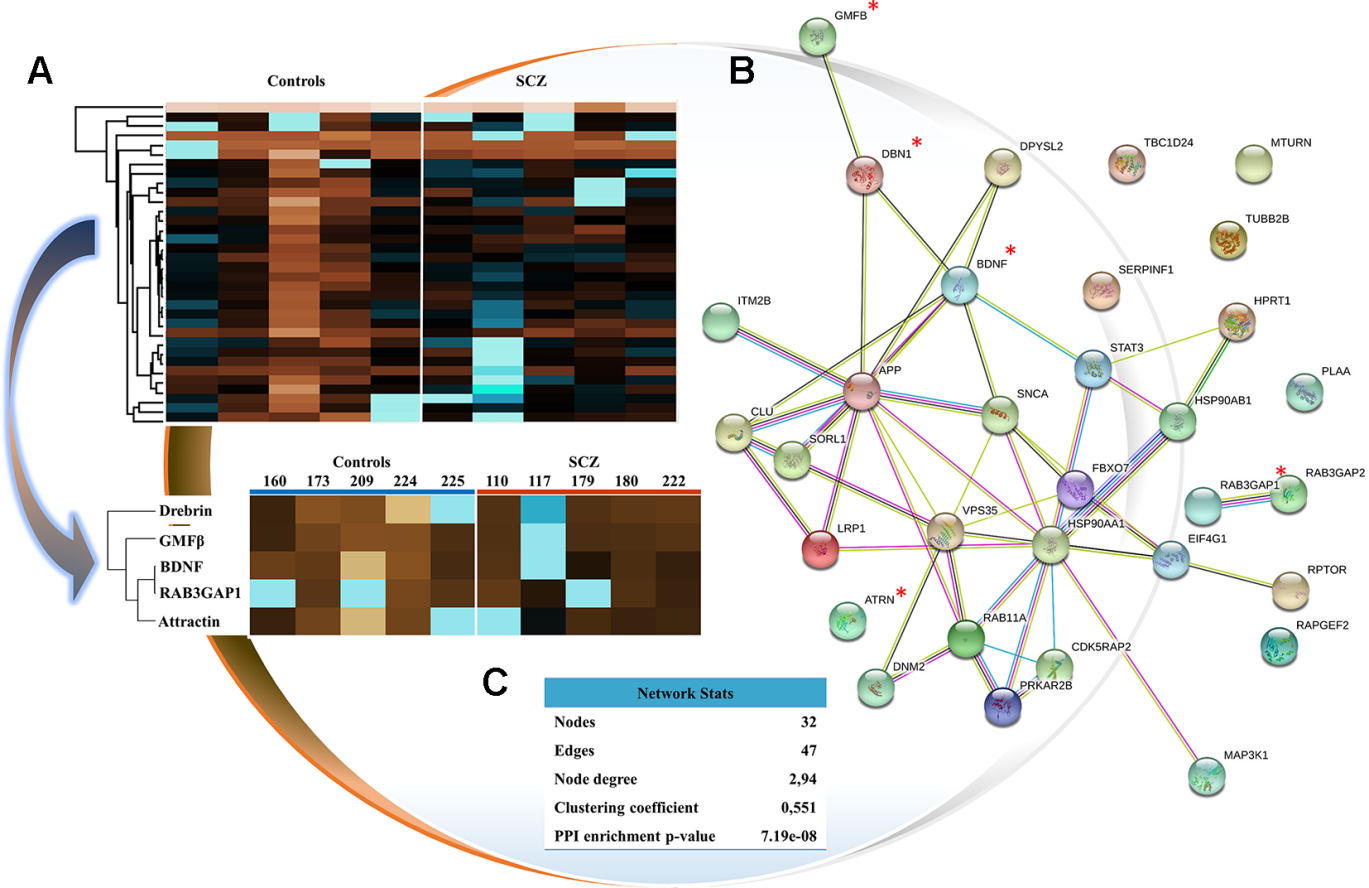
Analysis and characterization of selected proteins. **(A)** Heat-map representation of the proteins selected (34 proteins of interest) that converges on the mapping of the 5 proteins analyzed. The rows link the proteins according to their mean values (number of clusters = 300; maximum number of interactions = 10; and number of restarts = 1). Upregulated proteins are represented in blue, and downregulated proteins in brown. The numbering of the columns corresponds to our encoding of individuals. **(B)** Protein–protein interaction mapping by STRING tool. Study proteins are marked with a red asterisk. **(C)** STRING network analysis.

The available evidence suggests that schizophrenia causes a dysfunction in synaptic, neurotransmission, and neuronal patterns (20–22). Based on this, our analytical findings report other proteomic perspectives, founded on biological connections, protein networks, and specific pathways (**Figure 1**). Protein–protein interaction (PPI) maps were analyzed by STRING Interactome, which enabled us to understand protein networks and to identify a cluster of proteins involved in the coordination of the biological pathways previously mentioned. Thus, the STRING PPI network connectivity reported a *P* value = 7.19e^−8^, demonstrating that the proteins are at least partially biologically connected as a group. The characteristics of STRING network include number of nodes, number of edges, average node degree, average local clustering coefficient, and PPI enrichment *P* value (**Figure 1**). Of the total of 34 proteins screened, 5 proteins associated with psychoneuroimmune pathways in schizophrenia were analyzed, which could be potential new analytes: drebrin, GMF-β, BDNF, RAB3GAP1, and attractin. These proteins are involved in critical neurobiological processes closely linked to schizophrenia. Finally, heat-mapping of these five proteins proved the existence of a biological connection between them. In this map, upregulated proteins were represented in blue and downregulated proteins in brown (**Figure 1**).

**Figure 2:**
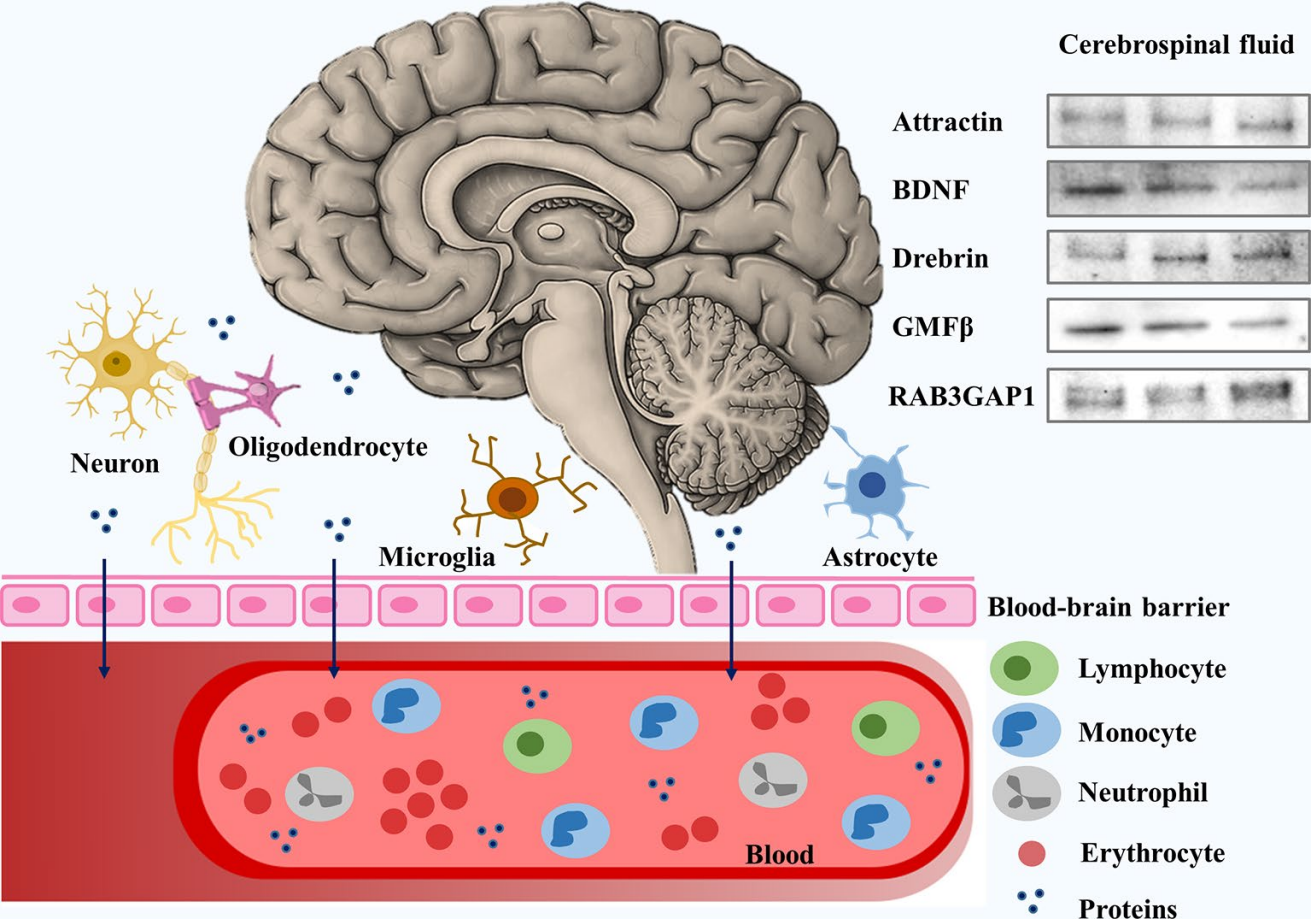
Schematic representation of central nervous system (CNS)–originated proteins analyzed in cerebrospinal fluid (CSF). Proteins involved in psychoneuroimmune pathways and CNS-originated are detected in CSF samples and pass through the blood–brain barrier into peripheral blood. BDNF, brain-derived neurotrophic factor; GMF-β, glia maturation factor beta; RAB3GAP1, 115-kDa isoform of Rab3 GTPase-activating protein catalytic subunit.

Psychoneuroimmune pathways are undoubtedly correlated with schizophrenia. We selected these proteins based on their functions on the CNS. However, to understand how CNS-originated proteins leaked to peripheral blood, we analyzed CSF samples of six controls by Western blot, proving that drebrin, GMF-β, BDNF, RAB3GAP1, and attractin are present in the CSF and can cross the blood–brain barrier (BBB), and can be found in peripheral blood (**Figure 2**). Moreover, we analyzed the correlation between levels of proteins in CSF and plasma samples of controls. Thus, we established that levels of proteins in CSF and plasma are comparable due to the lack of statistical significance (1.000 ± 0.10 vs. 1.000 ± 0.42, Pattractin = 0.9999; 1.000 ± 0.12 vs. 1.000 ± 0.39, PBDNF = 0.8205; 1.000 ± 0.20 vs. 1.000 ± 0.42, Pdrebrin = 0.9997; 1.000 ± 0.12 vs. 1.000 ± 0.49, PGMFβ = 0.8009; and 1.000 ± 0.13 vs. 1.000 ± 0.59, PRAB3GAP1 = 0.9999).

**Figure 3:**
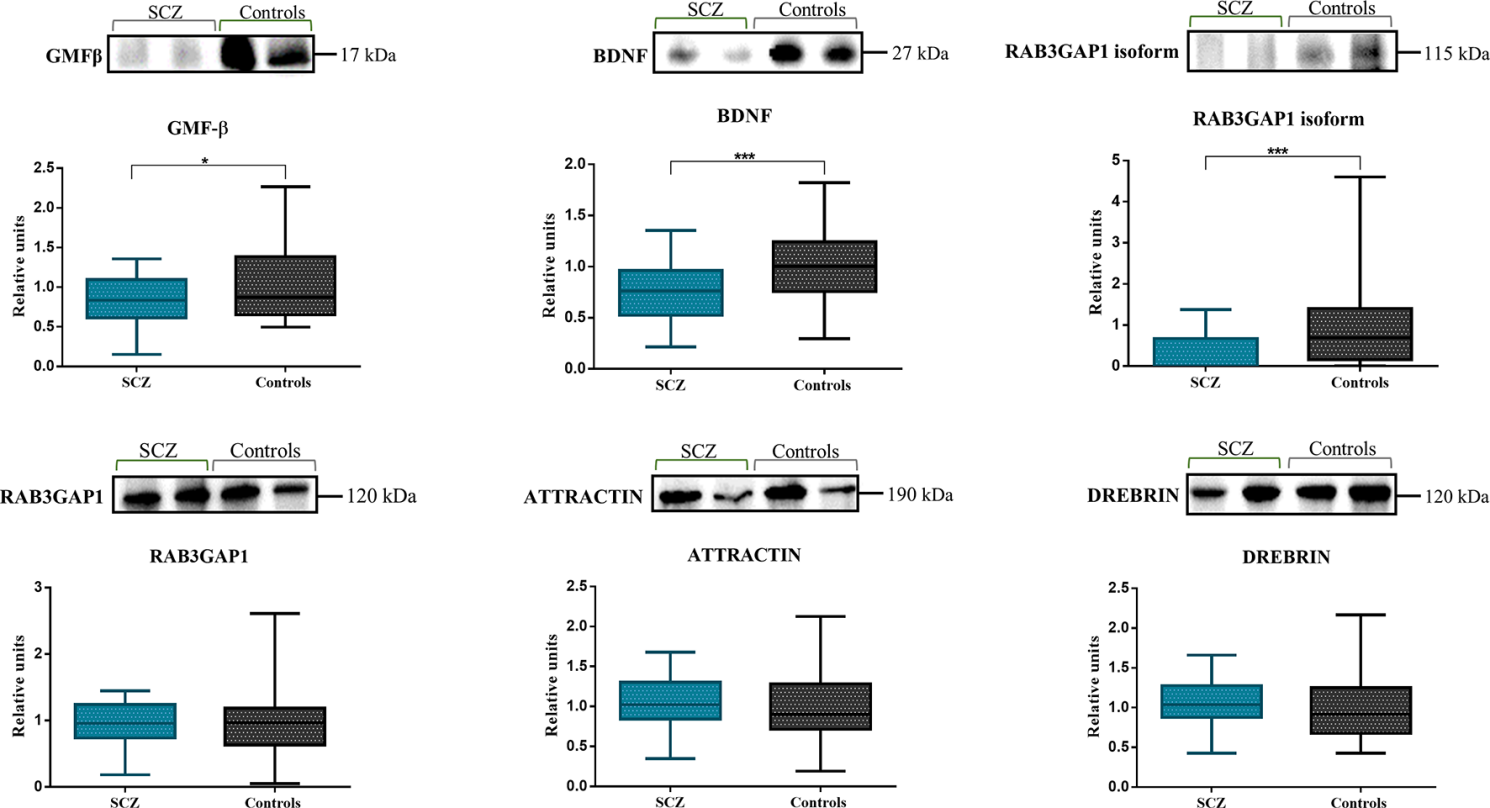
Western blot of the plasma of patients with schizophrenia and healthy controls. A significant decrease in the levels of GMF-β (*P* = 0.0256), BDNF (*P* = 0.0008), and the 115-kDa isoform of RAB3GAP1 (*P* = 0.0002) was observed in schizophrenic patients (n = 45) as compared to the controls (n = 43). No significant correlation was detected with the attractin (*P* = 0.5345), drebrin (*P* = 0.3917), and RAB3GAP1 (*P* = 0.6729) proteins. Relative units refer to the mean percentage of control values.

Thus, to verify the role of these five proteins in schizophrenia, we analyzed changes in plasma protein levels using the Western blot (**Figure 3**). The plasma levels of the selected proteins in both the patients with schizophrenia and the healthy controls were measured and calculated based on the mean percentage of control values. Upon quantification, this analysis revealed a significant decrease in the levels of GMF-β (0.8357 ± 0.30 vs. 1.000 ± 0.49, *P* = 0.0256), BDNF (0.7571 ± 0.30 vs. 1.000 ± 0.39, *P* = 0.0008), and the 115-kDa isoform of RAB3GAP1 (0.2717 ± 0.45 vs. 1.000 ± 1.07, *P* = 0.0002) in schizophrenic patients as compared to healthy volunteers. Moreover, the effect size (Hedges’ g) of GMF-β was 0.407 with a 95% confidence interval (CI) = −0.016–0.829; BDNF was 0.700 with a CI = 0.37–1.131; and 115-kDa isoform of RAB3GAP1 was 0.895 with a CI = 0.456–1.333. Attractin, drebrin, and the RAB3GAP1 all presented a *P* value >0.05 (1.052 ± 0.31 vs. 1.000 ± 0.42, *P* = 0.5345; 1.070 ± 0.29 vs. 1.000 ± 0.42, *P* = 0.3917; and 0.9547 ± 0.32 vs. 1.000 ± 0.59, *P* = 0.6729, respectively), thus revealing an absence of significant statistical correlation. The 115-kDa isoform of RAB3GAP1 was detected in Western blot analysis, and its presence was also confirmed in the Excel database generated by the Proteome Discoverer software (Rab3 GTPase-activating protein catalytic subunit isoform X1; *Homo sapiens*).

On the other hand, we subdivided the group of patients with schizophrenia into two groups: FES group and chronic schizophrenia group. Then, we analyzed changes in proteins levels of both groups of patients compared to healthy subjects (**Table 1**). One-way ANOVA test after *P*-value adjustment (Bonferroni post-hoc test) detected levels of BDNF statistically significant (F_(2,85)_ = 7.194, *P* = 0.0014) being lower in the FES group (0.6449 ± 0.24 vs. 1.000 ± 0.39, *P* = 0.0031) compared to chronic schizophrenia (0.7986 ± 0.32 vs. 1.000 ± 0.39, *P* = 0.0131). The 115-kDa isoform of RAB3GAP1 also presented relevant results (F_(2,85)_ = 7.918, *P* = 0.0008) with a statistical significance in FES group (0.1372 ± 0.43 vs. 1.000 ± 1.07, *P* = 0.0080) and chronic schizophrenia group (0.3166 ± 0.45 vs. 1.000 ± 1.07, *P* = 0.0020). The Bonferroni multiple comparisons test revealed a significant result in the GMF-β levels in the group of chronic schizophrenia (0.8066 ± 0.32 vs. 1.000 ± 0.49, *P* = 0.0453). Attractin, drebrin, and the RAB3GAP1 levels did not yield correlation with the severity of the disease (**Table 1**).

**Table 1:** One-way ANOVA and bonferroni multiple comparisons test results.

Parameter	FES	Chronic SCZ	Controls
**N**	**10**	**35**	**43**
**GMF-β** **^1^**	0.8877 ± 0.25	0.8066 ± 0.32	1.000 ± 0.49
P-value Bonferroni post-hoc test	0.5257	**0.0453** ^*^	
One-way ANOVA		F_(2,85)_ = 2.814, *P* = 0.0669	
**BDNF** **^1^**	0.6449 ± 0.24	0.7986 ± 0.32	1.000 ± 0.39
P-value Bonferroni post-hoc test	0.0031*	0.0131*	
One-way ANOVA		**F**_(2,85)_ ** = 7.194, ** *P* = 0.0014*	
**115-kDa isoform RAB3GAP1** **^1^**	0.1372 ± 0.43	0.3166 ± 0.45	1.000 ± 1.07
P-value Bonferroni post-hoc test	0.0080*	0.0020*	
One-way ANOVA		**F**_(2,85)_ ** = 7.918, ** *P* = 0.0008*	
**RAB3GAP1** **^1^**	0.9618 ± 0.31	0.9524 ± 0.33	1.000 ± 0.59
P-value Bonferroni post-hoc test	>0.9999	>0.9999	
One-way ANOVA		F_(2,85)_ = 0.0901, *P* = 0.9139	
**Drebrin** **^1^**	1.2494 ± 0.25	1.010 ± 0.28	1.000 ± 0.41
P-value Bonferroni post-hoc test	0.0993	>0.9999	
One-way ANOVA		F_(2,85)_ = 2.122, *P* = 0.1269	
**Attractin** **^1^**	1.2426 ± 0.31	0.9885 ± 0.29	1.000 ± 0.42
P-value Bonferroni post-hoc test	0.1238	>0.9999	
One-way ANOVA		F_(2,85)_ = 2.080, *P* = 0.1322	

One-way ANOVA followed by Bonferroni post-hoc test: comparison of patients groups with control group. *Statistical significance: P ≤ 0.05. ^1^Relative units of plasma levels (mean ± SD). FES, first-episode schizophrenia; SCZ, schizophrenia; GMF-β, glia maturation factor beta; BDNF, brain-derived neurotrophic factor; RAB3GAP1, Rab3 GTPase-activating protein catalytic subunit. N- number of participants.

We subsequently analyzed correlations between levels of proteins with statistical significance and symptomatology (PANSS scores), age of illness onset, and duration of illness (**Supplementary Table 2**). No statistical significance was found between the protein levels and these parameters.

## Discussion

Large-scale study of protein expression using high-resolution proteomics can be used for the discovery of disease biomarkers in patient plasma samples (23). The use of instruments such as high-resolution mass spectrometry (e.g. Orbitrap) together with enhancements in LFQ and the precise preparation of samples has allowed for improving protein detection with an excellent accuracy (24). In addition, it may be concluded that schizophrenia is a very complex disease that combines intrinsic and extrinsic factors responsible for synaptic deficits, neuronal dysfunctions, and immune and neurotransmission alterations (25–27). One of the handicaps in the detection and research of molecular brain events, such as synaptic activity, plasticity, neuroinflammation, or neurodegeneration, is how to detect these changes in patients with an active disease. To predict these cerebral events, we used deep proteomic techniques aimed at detecting proteins or changes in the patients’ plasma samples. Human plasma is the largest and deepest sample of the entire set of proteins that characterizes the proteome, containing the typical plasma proteins, additionally tissue proteins, and immunoglobulin sequences (28). In the last years, several studies have been analyzing the blood plasma and serum proteome of patients with schizophrenia (8, 29). Focusing in these studies, most are matched case–control studies whose sample size average oscillates between 30 patients with schizophrenia (cases group) and 32 healthy volunteers (control group) (30–38). Moreover, LFQ of proteins by LC-MS/MS is the most common technique to analyze the proteome whose range of proteins with altered abundance varies between 6 and 35 (15 proteins on average). Additionally, multiplex immunoassays have also been widely used; however, limitations of multiplexing itself or related to the specificity for protein isoforms make them incompatible with some investigations, whereby the MS-based proteomics becomes the preferred technique due to its unprecedented accuracy (29, 39). Finally, the main findings reported by these studies suggest alterations in inflammatory and immune response, coagulation cascade, glucose and lipid metabolism, and structural proteins (29). Despite the high sensitivity and specificity of LC/MS-MS approaches, none of the studies contrasted and validated their results with other techniques such as enzyme-linked immunosorbent assays (ELISA) or Western blot.

Synaptic deficits are associated with abnormal glial–neuronal connections, as well as with genetic, immunological, and environmental factors that may contribute to imbalances in synaptic homeostasis and, consequently, to a worsening of the symptoms of schizophrenia (20). Drebrin is an actin-binding protein that regulates synaptic plasticity and whose absence is associated with abnormal synaptic dysfunction (40). The BDNF protein is the most abundant neurotrophin in the CNS and a key regulator of synaptic plasticity (41). GMF-β, on the other hand, is correlated with glial and neural dysfunctions. This protein is expressed in the microglia, and in the CNS neurons, astrocytes, and oligodendrocytes, and is considered a growth and differentiation factor (42, 43). GMF-β is also associated with neuroinflammative and neurodegenerative disorders (43). Attractin is a soluble protein released by activating T lymphocytes, and linked to immune cell interaction (44). In the CNS, attractin contributes to progressive neurodegeneration associated to neuroinflammation where T lymphocytes release attractin, which results in neuronal cell death and hypomyelination (45, 46). Finally, RAB3GAP1 is a key regulator of the exocytosis of neurotransmitters and hormones (47). These five proteins regulate crucial processes involved in proper brain activity. Additionally, there is an association between these proteins and neuroinflammatory and neurodegenerative conditions such as multiple sclerosis and Alzheimer’s and Parkinson’s diseases (43, 48). For example, a decrease in BDNF expression was correlated with neuronal loss in neurodegenerative diseases, including Parkinson’s disease or Alzheimer’s disease (49, 50). On the other hand, RAB3GAP1 also plays an important role in the autophagic pathway that results in the intercellular accumulation of proteins, whose dysregulation is related with several conditions such as cancer and cardiovascular or neurodegenerative diseases (51). Moreover, other studies reveal a significant reduction of drebrin that may be a potential indicator of impaired dendritic arborization and synaptic dysfunction in neurodegenerative diseases as Alzheimer’s disease (52, 53). Regarding attractin, this protein is involved in neuroprotection through the regulation of myelination, neuroinflammation, and metabolism of reactive oxygen species (ROS) (45, 54). Thus, abnormal levels of attractin are associated with neurodegenerative processes (45). Finally, GMF modulates the glial activation induced by beta-amyloid, inflammatory cytokine production, and neuronal damage, whose upregulation of its expression generates inflammation in neurodegenerative diseases (55, 56).

In our study, we detected by LC-MS/MS that these proteins, specifically BDNF, GMF-β, and the 115-kDa isoform of RAB3GAP1, are downregulated in schizophrenia, with significantly reduced levels being detected in the plasma of schizophrenic patients. Additionally, we found a significant weight Hedges’ g of 0.407 for GMF-β; 0.700 for BDNF; and 0.895 for 115-kDa isoform of RAB3GAP1, whose results have shown a consistent effect and substantially significant. Specifically, levels of BDNF were lower in the FES group. It was described in FEP that there were found associations between reduced BDNF gene expression levels in severe mental disorders, associated with increased inflammation and smaller hippocampal volume (57). We corroborated by LC-MS/MS and Western blot that plasmatic BDNF is decreased in both FES and chronic schizophrenia. GMF-β protein is a protein implicated in differentiation of brain cells and stimulation of neural regeneration. We described that GMF-β plasmatic had decreased levels in the group of patients with chronic schizophrenia. Finally, RAB3GAP1 is a protein that participates in neurodevelopmental processes such as proliferation, migration, and differentiation before synapse formation with several isoforms. We described that levels of 115-kDa isoform of RAB3GAP1 were reduced in both groups. This may lead to a loss of brain functions associated with synaptic, neuronal, and neurotransmission deficits. The loss of grey matter may be explained by a reduction in the levels of synaptic activity, thus justifying the neurodevelopment hypothesis of schizophrenia (58). Synaptic plasticity is essential for memory and learning processes, and is involved in the complex task of neuronal organization within the brain (normal brain maturation) (59). Therefore, cognitive dysfunction in schizophrenia characterized as "dysconnectivity" involves abnormalities in synaptic plasticity, transmitter receptors (number, composition, and phosphorylation status), and neuronal structure (e.g. axonal fibers) (60).

Being able to find potential plasma biomarkers that are capable of predicting changes in synaptic pruning can open a new pathway for the diagnosis and treatment of schizophrenia. Starting from the analysis by LC-MS/MS, we predict five candidates that may be relevant to demonstrate alterations between the clinical development of schizophrenia and plasma. Although the expression of these five proteins is broad, the main place of expression of all of them is the CNS. To demonstrate that these five proteins are able to bind to the BBB and reach the plasma, we analyze their presence in CSF samples (**Figure 2**).

Regarding the disease’s clinical symptomatology, schizophrenic patients that participated in our study experienced more pronounced negative symptoms (**Table 2**). The available evidence suggests that negative symptoms are a relevant multidimensional construct in both chronic schizophrenia and FES (61). The presence of negative symptoms at early stages of the disease associated with poor pre-morbid adjustment is a predictive factor for medium-term severe negative symptoms (62). Furthermore, psychopathological models report impaired insight in relation to the severity of the positive symptoms, as their intensity tends to fluctuate with each psychotic episode, although positive symptomatology tends to improve over time (as opposed to negative symptomatology) with antipsychotic treatment (63). Moreover, cognitive dysfunction starts during adolescence, and its onset is accompanied by the first signs of schizophrenia worsening with chronicity of the disease (64). However, no statistical significance was found between the levels of the studied proteins and the PANSS scores.

**Table 2:** Demographic and Clinical Data.

Parameter	SCZ	Controls	P value
*Total sample size (N)*	45	43	
*Age (mean ± SD)*	40.78 ± 15.04	43.82 ± 14.42	0.4593^1^
*Sex (M/F)*	28/17	26/17	1.0000^2^
*Age of illness onset (mean ± SD)*	21.63 ± 16.07	–	–
*Duration of illness (mean ± SD)*	11.63 ± 10.77	–	–
**PANSS** * (mean ± SD)*			
**PANSS** *Positive*	19.78 ± 6.78	–	–
*PANSS Negative*	28.73 ± 8.38	–	–
*PANSS General*	33.75 ± 8.35	–	–
*PANSS Total*	82.25 ± 18.22	–	–

^1^Mann–Whitney U test P value; ^2^Fisher’s exact test P value. SCZ, schizophrenia; PANSS, Positive and Negative Syndrome Scale; PANSS positive and negative ranges, 7–49; PANSS general range, 16–112; PANSS total range, 30–210 (16). Statistical significance, P ≤0.05.

Our study has some limitations that should be mentioned. First of all, our sample size with regard to the FES group is small, which consequently limits its statistical power and warrants the need for future research with a larger number of FES patients to achieve robust evidence. Secondly, our analysis did not consider the difference between antipsychotic drugs. However, the fact is that the patients were admitted to the psychiatric ward allowing the control of the treatment adherence. Despite these limitations, this study yielded significant results that allowed for channeling the search of an analyte panel for schizophrenia. In conclusion, there is a great potential for BDNF, GMF-β, and the 115-kDa isoform of RAB3GAP1 proteins to act as possible biomarkers of schizophrenia, and they may even differentiate early stages (e.g. BDNF) from later stages (e.g. GMF-β) of the disease.

Cognitive disorders and neuroinflammatory phenomena occur years before the FES, associated with an increase in the synthesis of dopamine in the striatum (64). Our study opens the door to detecting cognitive alterations in a simple way in plasma samples. Being able to measure potential biomarkers of cognitive status in peripheral samples opens up a very novel way of research in the treatment and diagnosis of schizophrenia, which in the future would greatly improve patient quality of care.

## Data Availability Statement

The raw data supporting the conclusions of this manuscript will be made available by the authors, without undue reservation, to any qualified researcher.

## Ethics Statement

The studies involving human participants were reviewed and approved by Galician Network of Research Ethics Committees. The patients/participants provided their written informed consent to participate in this study.

## Author Contributions

DR-A, TR-B, and CS designed the study and acquired data to analysis. DR-A and CS wrote the article. CR-J, MV-C, EH, AC and JO made substantial contributions to conception and design of the study. All authors read and approved the final version of the manuscript

## Funding 

This work was financially backed by the Foundation for Science and Technology (FCT, Fundação para a Ciência e Tecnologia) within the framework of grant SFRH/BD/135623/2018 awarded to Daniela Rodrigues- Amorim, and another grant of Fundación Tatiana Pérez de Guzmanel Bueno provided to Carlos Spuch. Our research was further supported by the Carlos III Health Institute (ISCIII, Instituto Carlos III) through grant P16/00405 and co-funding awarded by the Spanish Foundation of Rare Diseases (FEDER, Fundación Española de Enfermedades Raras) to José Manuel Olivares.

## Conflict of Interest

The authors declare that the research was conducted in the absence of any commercial or financial relationships that could be construed as a potential conflict of interest.

## Supplementary Material

Supplementary Figure 1 Enrichment analysis of selected proteome results.
