# Supplementary figures and images for: Proteomics in Schizophrenia: A Gateway to Discover Potential Biomarkers of Psychoneuroimmune Pathways
Source: Front Psychiatry. 2019 Nov 29;10:885. doi: 10.3389/fpsyt.2019.00885 (PMC6897280; doi:10.3389/fpsyt.2019.00885)

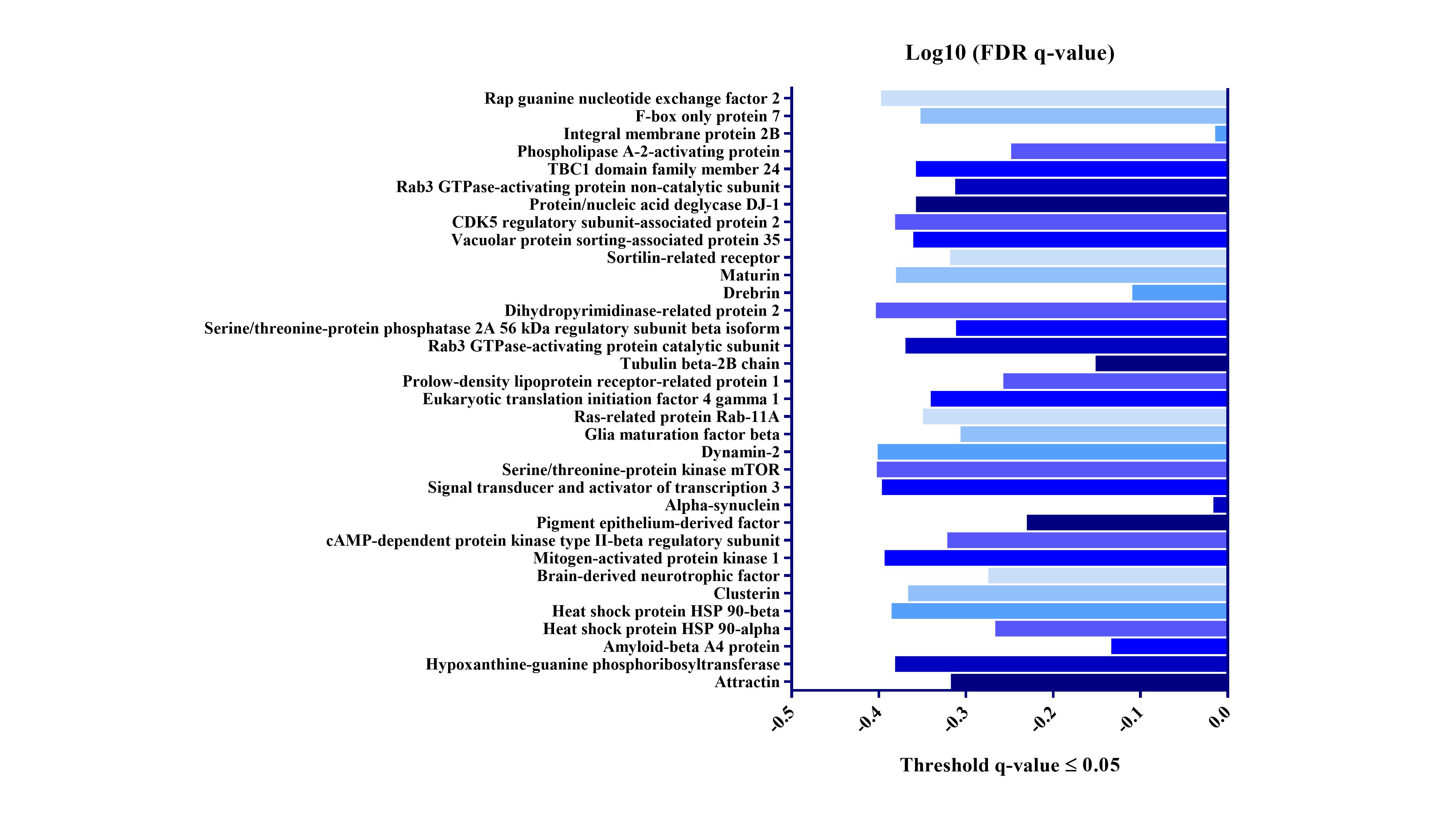

Supplement: Supplementary Figure 1 — Enrichment analysis of selected proteome results. [file Image_1.tif]
