# Supplementary material for: Proteomics in Schizophrenia: A Gateway to Discover Potential Biomarkers of Psychoneuroimmune Pathways
Source: Front Psychiatry. 2019 Nov 29;10:885. doi: 10.3389/fpsyt.2019.00885 (PMC6897280; doi:10.3389/fpsyt.2019.00885)
Supplement: Supplementary file 2 [file Table_1.docx]

**Supplementary Table 1:** Functional proteins screened based on psychoneuroimmune pathways using the UniProtKB/Swiss-Prot.

| **Proteins name** | **Gene** | **Activity (molecular and biological functions)** | **Location** | **Protein ID** | **Log10**  **(FDR *Q* value)** |
| --- | --- | --- | --- | --- | --- |
| **Attractin** | *ATRN* | It is involved in initial immune cell clustering during inflammatory response. It plays a critical role in normal myelination in the CNS, cerebellum development, myelination, and response to oxidative stress. | Plasma membrane, extracellular | O75882 | -0.315 |
| **Hypoxanthine-guanine phosphoribosyltransferase** | *HPRT1* | It converts guanine to guanosine monophosphate, and hypoxanthine to inosine monophosphate, and is involved in CNS neuron development, cerebral cortex neuron differentiation, dendrite morphogenesis, dopamine metabolic processes, and the positive regulation of dopamine metabolic processes. | Cytoplasm | P00492 | -0.379 |
| **Amyloid-beta A4 protein** | *APP* | It functions as a cell surface receptor and performs physiological functions on the surface of neurons relevant to neurite growth, neuronal adhesion and axonogenesis. | Membrane | P05067 | -0.131 |
| **Heat shock protein HSP 90-alpha** | *HSP90AA1* | It functions as a cell surface receptor and performs physiological functions on the surface of neurons relevant to neurite growth, neuronal adhesion and axonogenesis. | Membrane | P05067 | -0.246 |
| **Heat shock protein HSP 90-beta** | *HSP90AB1* | It functions as a molecular chaperone that promotes the maturation, structural maintenance and proper regulation of specific target proteins involved, for instance, in cell cycle control and signal transduction, as well as Tau protein binding and CNS neuron axonogenesis | Nucleus, plasma membrane | P07900 | -0.383 |
| **Clusterin** | *CLU* | Isoform 1 of this protein functions as an extracellular chaperone that prevents aggregation of non-native proteins. It is also involved in microglial cell activation, microglial cell proliferation, and the positive regulation of amyloid-beta formation. | Extracellular, endoplasmic reticulum, cytosol, nucleus | P10909 | -0.364 |
| **Brain-derived neurotrophic factor** | *BDNF* | During development, it promotes the survival and differentiation of selected neuronal populations of the peripheral and central nervous system. It participates in axonal growth, pathfinding and the modulation of dendritic growth and morphology. It is a major regulator of synaptic transmission and plasticity in adult synapses at many regions of the CNS. The versatility of BDNF is emphasized by its contribution to a range of adaptive neuronal responses including LTP, LTD, certain forms of short-term synaptic plasticity, as well as homeostatic regulation of intrinsic neuronal excitability. | Extracellular | P23560 | -0.272 |
| **Mitogen-activated protein kinase 1** | *MAPK1* | Serine/threonine kinase acts as an essential component of the MAP kinase signal transduction pathway. MAPK1/ERK2 and MAPK3/ERK1 are the 2 MAPKs playing the most important role in the MAPK/ERK cascade. It is also involved in cellular response to dopamine, as well as in learning or memory processes. | Nucleus, cytoskeleton | P28482 | -0.391 |
| **cAMP-dependent protein kinase type II-beta regulatory subunit** | *PRKAR2B* | The regulatory subunit of the cAMP-dependent protein kinases is involved in cAMP signaling in cells. Type II regulatory chains mediate membrane association by binding to anchoring proteins, including the MAP2 kinase. It is also involved in learning processes and the response to clozapine. | Plasma membrane | P31323 | -0.319 |
| **Pigment epithelium-derived factor** | *SERPINF1* | As a neurotrophic protein, it induces extensive neuronal differentiation in retinoblastoma cells. Since it does not undergo the S (stressed) to R (relaxed) conformational transition characteristic of active serpins, it exhibits no serine protease inhibitory activity. It is also involved in the negative regulation of neuron death, the positive regulation of neurogenesis, and the positive regulation of neuron projection development. | Extracellular | P36955 | -0.228 |
| **Alpha-synuclein** | *SNCA* | It may be involved in the regulation of dopamine release and transport. It induces fibrillization of microtubule-associated Tau proteins, and it reduces neuronal responsiveness to various apoptotic stimuli, thus leading to a decreased caspase-3 activation. | Cytosol, nucleus, extracellular | P37840 | -0.014 |
| **Signal transducer and activator of transcription 3** | *STAT3* | It acts as a signal transducer and transcription activator that mediates cellular responses to interleukins, KITLG/SCF, LEP and other growth factors. It is also involved in astrocyte differentiation, cytokine-mediated signaling pathways, the negative regulation of neuron death, and the negative regulation of neuron migration. | Nucleus | P40763 | -0.394 |
| **Serine/threonine-protein kinase mTOR** | *MTOR* | This is a serine/threonine protein kinase acting as a central regulator of cellular metabolism, growth and survival in response to hormones, growth factors, nutrients, energy and stress signals. MTOR directly or indirectly regulates the phosphorylation of at least 800 proteins. It is also involved in the positive regulation of neuron death, the positive regulation of neuron maturation, and the positive regulation of the nitric oxide biosynthetic process. | Endoplasmic reticulum, nucleus, Golgi apparatus | P42345 | -0.400 |
| **Dynamin-2** | *DNM2* | This microtubule-associated force-producing protein is involved in the production of microtubule bundles and is capable of binding and hydrolyzing GTP. It plays a significant role in the regulation of neuron morphology, axon growth, the formation of neuronal growth cones, cellular response to dopamine, neuron projection morphogenesis, and the positive regulation of the nitric oxide biosynthetic process. | Plasma membrane, cytoskeleton | P50570 | -0.399 |
| **Glia maturation factor beta** | *GMFB* | This protein causes differentiation of brain cells, stimulation of neural regeneration, and inhibition of the proliferation of tumor cells. It is also involved in the learning processes and the development of the nervous system. | Intracellular | P60983 | -0.304 |
| **Ras-related protein Rab-11A** | *RAB11A* | The small GTPases Rab proteins are key regulators of intracellular membrane trafficking, from the formation of transport vesicles to their fusion with membranes. Rabs cycle between an inactive GDP-bound form and an active GTP-bound form that is capable of recruiting different sets of downstream effectors directly responsible for vesicle formation, movement, tethering and fusion, to the membrane. It is also involved in neuron projection development, exosomal secretion, neurotransmitter receptor transport, endosome to postsynaptic membrane regulation, and the positive regulation of axon extension. | Plasma membrane, endosome | P62491 | -0.347 |
| **Eukaryotic translation initiation factor 4 gamma 1** | *EIF4G1* | It is a component of protein complex eIF4F involved in the recognition of the mRNA cap, ATP-dependent unwinding of the 5'-terminal secondary structure and the recruitment of mRNA to the ribosome. It is also involved in the negative regulation of neuron death, the positive regulation of neuron differentiation, the regulation of polysome binding, the regulation of presynaptic assembly, translation, the regulation of cellular response to stress, and the positive regulation of peptidyl-serine phosphorylation. | Cytosol, nucleus | Q04637 | -0.338 |
| **Prolow-density lipoprotein receptor-related protein 1** | *LRP1* | It is an endocytic receptor involved in endocytosis and the phagocytosis of apoptotic cells. It is required for early embryonic development and may modulate cellular events, such as APP metabolism, kinase-dependent intracellular signaling, neuronal calcium signaling and neurotransmission. It is also involved in ageing, amyloid-beta clearance, astrocyte activation involved in the immune response, cellular response to amyloid-beta, cerebral cortex development, the negative regulation of neuron projection development, and the positive regulation of amyloid-beta clearance. | Plasma membrane | Q07954 | -0.225 |
| **Tubulin beta-2B chain** | *TUBB2B* | Tubulin is the major constituent of microtubules. It plays a critical role in proper axon guidance in both central and peripheral axon tracts, and participates in neuronal migration. | Cytoskeleton | Q9BVA1 | -0.149 |
| **Rab3 GTPase-activating protein catalytic subunit** | *RAB3GAP1* | It is a probable catalytic subunit of a GTPase-activating protein with specificity for the Rab3 subfamily (RAB3A, RAB3B, RAB3C and RAB3D). Rab3 proteins are involved in the regulated exocytosis of neurotransmitters and hormones. It is required for normal eye and brain development and may also participate in neurodevelopmental processes such as presynaptic proliferation, migration and differentiation, and non-synaptic vesicular release of neurotransmitters. | Cytoplasm | Q15042 | -0.367 |
| **Serine/threonine-protein phosphatase 2A 56 kDa regulatory subunit beta isoform** | *PPP2R5B* | As the regulatory component of the PP2A holoenzyme, it modulates substrate specificity, subcellular localization, and responsiveness to phosphorylation. Its phosphorylated form mediates the interaction between PP2A and AKT1, leading to AKT1 dephosphorylation, the positive regulation of neurotrophin TRK receptor signaling pathway, and the positive regulation of neuron projection development. | Cytoplasm | Q15173 | -0.309 |
| **Dihydropyrimidinase-related protein 2** | *DPYSL2* | It plays a role in neuronal development and polarity, as well as in axon growth and guidance, neuronal growth cone collapse and cell migration. It is required for signaling by class-3 semaphorins and subsequent remodeling of the cytoskeleton. It may also play a role in endocytosis, axon guidance, brain development, nervous system development, and regulation of axon extension. | Cytosol, cytoskeleton | Q16555 | -0.401 |
| **Drebrin** | *DBN1* | Drebrin might play a role in cell migration, the extension of neuronal processes and dendrite plasticity. It is a neural precursor of cell proliferation involved in the regulation of dendrite development and neuronal synaptic plasticity. | Cytoplasm, cell cortex, cell junction | Q16643 | -0.107 |
| **Maturin** | *MTURN* | It may be involved in early neuronal development. As a developmental protein, maturin is required for normal primary neurogenesis. | Intracellular | Q8N3F0 | -0.378 |
| **Sortilin-related receptor** | *SORL1* | It is likely to be a multifunctional endocytic receptor that may participate in the uptake of lipoproteins and proteases. It could also play a role in cell-cell interaction, the negative regulation of amyloid-beta formation, the negative regulation of neurogenesis, the negative regulation of neuron death, and the negative regulation of MAP kinase activity. | Golgi apparatus, endosome, extracellular, membrane | Q92673 | -0.316 |
| **Vacuolar protein sorting-associated protein 35** | *VPS35* | It acts as a component of the CSC. The CSC is believed to be the core functional component of retromer or respective retromer complex variants acting to prevent mis-sorting of selected transmembrane cargo proteins in the lysosomal degradation pathway. The recruitment of the CSC to the endosomal membrane involves RAB7A and SNX3. In addition, it is also involved in D1 dopamine receptor binding, the negative regulation of neuron death, the neurotransmitter receptor transport, the endosome to plasma membrane, the positive regulation of the dopamine receptor signaling pathway, and the regulation of presynaptic assembly. | Endosome | Q96QK1 | -0.358 |
| **CDK5 regulatory subunit-associated protein 2** | *CDK5RAP2* | It is a potential regulator of CDK5 activity via its interaction with CDK5R1. It is also a negative regulator of centriole disengagement (licensing) by maintaining centriole engagement and cohesion. In addition, it is involved in the regulation of mitotic spindle orientation and plays a role in neurogenesis, brain development, the negative regulation of neuron differentiation, and the regulation of neuron differentiation. | Cytoskeleton, golgi apparatus | Q96SN | -0.379 |
| **Protein/nucleic acid deglycase DJ-1** | *PARK7* | It plays a role in the regulation of the expression or stability of mitochondrial uncoupling proteins SLC25A14 and SLC25A27 in dopaminergic neurons of the substantia nigra pars compacta, and it attenuates the oxidative stress induced by the entry of calcium into the neurons via L-type channels during pace-making. Moreover, it participates in the negative regulation of hydrogen peroxide-induced neuron death, the negative regulation of hydrogen peroxide-induced neuron intrinsic apoptotic signaling pathway, the negative regulation of the neuron apoptosis process, and the positive regulation of the dopamine biosynthetic process. | Nucleus, plasma membrane, mitochondrion | Q99497 | -0.355 |
| **Rab3 GTPase-activating protein non-catalytic subunit** | *RAB3GAP2* | It is a regulatory subunit of a GTPase-activating protein with specificity for the Rab3 subfamily (RAB3A, RAB3B, RAB3C and RAB3D). Rab3 proteins are involved in the regulated exocytosis of neurotransmitters and hormones. The Rab3 GTPase-activating complex specifically converts active Rab3-GTP to the inactive form, Rab3-GDP. It is required for normal eye and brain development and may participate in neurodevelopmental processes such as proliferation, migration and differentiation before synapse formation, and in the non-synaptic vesicular release of neurotransmitters. | Cytoplasm | Q9H2M9 | -0.310 |
| **TBC1 domain family member 24** | *TBC1D24* | It may act as a GTPase-activating protein for proteins of the Rab family. It is also involved in the development of neuronal projections, probably mediated by a negative modulation of the ARF6 function. | Cytoplasm | Q9ULP9 | -0.355 |
| **Phospholipase A-2-activating protein** | *PLAA* | It positively regulates cytosolic and calcium-independent phospholipase A2 activity in a tumor necrosis factor alpha (TNF-alpha) or LPS-dependent manner, and, therefore, prostaglandin E2 biosynthesis. It also participates in the positive regulation of neuron migration, the positive regulation of dendrite extension, and the positive regulation of synaptic vesicle recycling. | Nucleus | Q9Y263 | -0.246 |
| **Integral membrane protein 2B** | *ITM2B* | It plays a regulatory role in the processing of the amyloid-beta A4 precursor protein and acts as an inhibitor of the amyloid-beta peptide aggregation and fibrils deposition. It also participates in the induction of neurite outgrowth, the negative regulation of amyloid precursor protein biosynthetic process, and the development of the nervous system. | Golgi apparatus, endosome, plasma membrane | Q9Y287 | -0.012 |
| **F-box only protein 7** | *FBXO7* | It is a substrate recognition component of a SCF E3 ubiquitin-protein ligase complex that acts as a mediator of the ubiquitination and subsequent proteasomal degradation of target proteins. It promotes MFN1 ubiquitination, the negative regulation of hydrogen peroxide-induced neuron death and the regulation of neuron projection development. | Cytosol, nucleus, mitochondrion | Q9Y3I1 | -0.350 |
| **Rap guanine nucleotide exchange factor 2** | *RAPGEF2* | It is involved in neuron migration and the formation of the major forebrain fiber connections forming the corpus callosum, the anterior commissure and the hippocampal commissure during brain development. It also participates in NGF-induced sustained activation of Rap1 at late endosomes and in the BDNF-induced axon outgrowth of hippocampal neurons. | Plasma membrane, endosome | Q9Y4G8 | -0.395 |

**Legend:** HSP: heat shock protein; AMP: adenosine monophosphate; mTOR: mammalian target of rapamycin; GTP: guanosine triphosphate; GDP: guanosine diphosphate; CDK5: cyclin dependent kinase 5; TBC: Tre-2/Bub2/Cdc16; CNS: central nervous system; PP2A: serine/threonine-protein phosphatase 2A; LTP: long-term potentiation; LTD: long-term depression; AKT: serine/threonine kinase; TRK: tyrosine kinase; CSC: retromer cargo-selective complex; SNX: sorting nexins; TNF: tumor necrosis factor; LPS: lipopolysaccharide; SCF: SKP-cullin, F-box containing complex; NGF: neuronal growth factor; BDNF: brain-derived neurotrophic factor
