# Supplementary material for: Proteomics in Schizophrenia: A Gateway to Discover Potential Biomarkers of Psychoneuroimmune Pathways
Source: Front Psychiatry. 2019 Nov 29;10:885. doi: 10.3389/fpsyt.2019.00885 (PMC6897280; doi:10.3389/fpsyt.2019.00885)
Supplement: Supplementary file 3 [file Table_2.docx]

**Supplementary Table 2:** Correlation analysis.

| ***Parameters*** | ***GMF-β*** | | ***BDNF*** | | ***RAB3GAP1 115kDa*** | |
| --- | --- | --- | --- | --- | --- | --- |
|  | ***r*** | ***P*** | ***r*** | ***P*** | ***r*** | ***P*** |
| *PANSS Positive* | 0.2308 | 0.1756 | -0.2180 | 0.1949 | -0.1821 | 0.2607 |
| *PANSS Negative* | -0.0296 | 0.8641 | -0.0169 | 0.9209 | 0.0644 | 0.6932 |
| *PANSS General* | 0.0718 | 0.6775 | -0.2705 | 0.1054 | -0.2333 | 0.1474 |
| *Illness onset* | 0.0343 | 0.8427 | 0.1528 | 0.3665 | 0.1628 | 0.3156 |
| *Duration of illness* | -0.0168 | 0.9228 | 0.0354 | 0.8356 | -0.1280 | 0.4312 |

**Legend:** Statistically significant according to Pearson’s correlation coefficients: *P* ≤0.05* (two-tailed). PANSS: Positive and Negative Syndrome Scale. GMF-β: glia maturation factor beta; BDNF: brain-derived neurotrophic factor; RAB3GAP1: Rab3 GTPase-activating protein catalytic subunit.
